# Oxygen and Glucose Deprivation Induces Bergmann Glia Membrane Depolarization and Ca^2+^ Rises Mainly Mediated by K^+^ and ATP Increases in the Extracellular Space

**DOI:** 10.3389/fncel.2017.00349

**Published:** 2017-11-03

**Authors:** Romain Helleringer, Oana Chever, Hervé Daniel, Micaela Galante

**Affiliations:** ^1^Pharmacology and Biochemistry of the Synapse, Institut des Neurosciences Paris-Saclay, Université Paris-Saclay, Université Paris-Sud, CNRS, UMR 9197, Orsay, France; ^2^Neuroglial Interactions in Cerebral Physiopathology, Center for Interdisciplinary Research in Biology, Collège de France, CNRS, UMR 7241, INSERM U1050, Labex Memolife, PSL Research University Paris, Paris, France

**Keywords:** glial cells, brain ischemia, patch clamp, calcium imaging, cerebellum

## Abstract

During brain ischemia, intense energy deficiency induces a complex succession of events including pump failure, acidosis and exacerbated glutamate release. In the cerebellum, glutamate is the principal mediator of Purkinje neuron anoxic depolarization during episodes of oxygen and glucose deprivation (OGD). Here, the impact of OGD is studied in Bergmann glia, specialized astrocytes closely associated to Purkinje neurons. Patch clamp experiments reveal that during OGD Bergmann glial cells develop a large depolarizing current that is not mediated by glutamate and purinergic receptors but is mainly due to the accumulation of K^+^ in the extracellular space. Furthermore, we also found that increases in the intracellular Ca^2+^ concentration appear in Bergmann glia processes several minutes following OGD. These elevations require, in an early phase, Ca^2+^ mobilization from internal stores via P2Y receptor activation, and, over longer periods, Ca^2+^ entry through store-operated calcium channels. Our results suggest that increases of K^+^ and ATP concentrations in the extracellular space are primordial mediators of the OGD effects on Bergmann glia. In the cerebellum, glial responses to energy deprivation-triggering events are therefore highly likely to follow largely distinct rules from those of their neuronal counterparts.

## Introduction

The structural and functional integrity of the brain is strictly dependent on the energy supply originating from continuous blood irrigation.

Glucose and oxygen availability can be severely compromised during ischemia, with multifaceted consequences on tissue health that develop gradually along an ischemic episode. One of the primary effects of ischemia is a decrease of metabolic ATP concentrations. The deriving inhibition of ATP-ases activity alters ionic concentration gradients, in particular leading to accumulation of both K^+^ and neurotransmitters in the extracellular space and to intracellular Ca^2+^ increases, events that can conjointly induce cell death (Rossi et al., 2007; Brouns and De Deyn, 2009).

Over recent years evidence has been accumulating involving glial cells in cerebral ischemia. On the one hand astrocytes are deemed to play a neuroprotective role as long-lasting glycogen stores, growth factors secreting elements and antioxidant agents (Nedergaard and Dirnagl, 2005; Rossi et al., 2007). Alternatively, astrocytes have also been found to contribute to tissue damaging by limiting the regeneration of injured axons through the glial scar (Silver and Miller, 2004; Pekny and Nilsson, 2005), by releasing toxic amounts of radicals (Gibson et al., 2005) and/or by contributing to brain tissue swelling (Kimelberg, 2005; Liang et al., 2007).

Overall, the exact role of astrocytes in the complex succession of pathological events following an ischemic episode still remains elusive. A full understanding of the mechanisms underlying ischemic responses in astrocytes is thus fundamental to provide new insight in ischemia pathology.

In the cerebellum, anoxic depolarizations are observed in Purkinje cells during Oxygen and Glucose Deprivation (OGD) episodes (Hamann et al., 2005; Mohr et al., 2010). These are triggered mainly by AMPA receptor activation following both glutamate exocytosis, reversal of glutamate transporters (Hamann et al., 2005) and H^+^-dependent glial glutamate release (Beppu et al., 2014). The impact of an ischemic event on cerebellar astrocytes has not been studied until now. In particular, Bergmann glial cells are radial astrocytes anatomically and functionally associated to Purkinje neurons. Their processes are closely juxtapposed to Purkinje cell spines (Xu-Friedman et al., 2001; Castejón et al., 2002) thus contributing to glutamate uptake (Bergles et al., 1997; Clark and Barbour, 1997; Takayasu et al., 2005) and to extracellular K^+^ and water homeostasis (Hirrlinger et al., 2008; Wang et al., 2012).

In view of their pivotal role in cerebellar physiology, we here focus on the impact of ischemia on Bergmann glial cells. We used a well-established model of OGD (Rossi et al., 2000), in *in vitro* cerebellar slices. Our results show that Bergmann glia respond to OGD with reversible membrane depolarizations and sustained intracellular Ca^2+^ increases. Interestingly, glutamate released during OGD has only minor effects on Bergmann glia, whereas extracellular ATP increases elicit Ca^2+^ mobilizations from internal stores. Finally, using K^+^-sensitive microelectrodes we show that Bergmann glia membrane depolarizations at the beginning of OGD are due to increases in extracellular K^+^ concentration while in a later phase, extracellular K^+^ accumulation is accompanied by the outflow of anions through DIDS-sensitive channels.

Our results provide important insight into the cellular mechanisms accompanying ischemic injuries to brain structures, and suggest a clear divergence between neuronal and glial OGD-related responses in the cerebellum.

## Materials and Methods

### Preparation of Cerebellar Slices

All experiments were conducted in accordance with the guidelines established by the European Communities Council Directive (2010/63/EU Council Directive Decree) and following the Annex IV of the French Decree (1st February 2013) establishing the guidelines for euthanasia. Experimental protocols were approved by the Animal welfare body of our Institution (Institut des Neurosciences, NeuroPSI). All efforts were made to minimize animal suffering and to reduce the number of animal used in the study.

Cerebellar slices were prepared from C57Bl/6J male mice or P2X7R knockout mice (P2X7R^−/−^, Pfizer), 2–3 month old. Animals were anesthetized by 2-bromo-2Cloro-1,1,1-trifluoroéthane (Sigma-Aldrich, France) before decapitation. Parasagittal cerebellar slice (250 μm) were obtained from the vermis with vibratome Microm HM 650V in an ice-cold Bicarbonate Buffered Solution (BBS) saturated with 5% CO_2_ and 95% O_2_ and supplemented with APV (50 μM) to prevent glutamate excitotoxicity during slicing. The composition of BBS is (in mM): 124 NaCl, 3 KCl, 1.15 KH_2_PO4, 1.15 MgSO_4_, 24 NaHCO_3_, 10 Glucose, 2 CaCl_2_ (osmolarity: 330 mOsm et pH 7.35). Slices were kept in BBS at room temperature then placed in the recording chamber and continuously superfused with BBS at a rate of 2.5 ml/min. Experiments were carried out at a temperature comprised between 29°C and 31°C. OGD was obtained substituting 10 mM glucose with 10 mM sucrose in the BBS in order to keep constant the osmolarity of the solution. Moreover the oxygen was replaced by nitrogen, this solution was then bubbled with 95% N_2_ and 5% CO_2_ gas mixture.

### Electrophysiology

Single-cell patch-clamp whole-cell recordings were performed with an Axopatch 200 amplifier. Patch pipettes were pulled from borosilicate glass capillaries with a horizontal puller and have a resistance of 5–7 MΩ when filled with the following intracellular solution (mM): K-gluconate 140, MgCl_2_ 1, KCl 4, Hepes 10, EGTA 0.75, Na_2_ATP 4, NaGTP 0.4 (osmolarity 300 mosm and pH 7.35). The stability of the series resistance was routinely checked by delivering brief (150 ms), hyperpolarizing pulses (10 mV). Recordings were interrupted when the series resistance increased by more than 20% of the initial value and this parameter was always compensated in recordings from Purkinje neurons. In current-clamp recordings, Bergmann glia membrane potential was measured without any current injection. In voltage-clamp experiments, Bergmann cells were held at −70 mV and Purkinje neurons at −60 mV. Liquid junction potential was not compensated. For double patch clamp experiments cells were recorded with and an Axopatch 200 and Axopatch 200B amplifiers. The I_OGD_ charge was calculated as the integral of the current (baseline adjusted to zero) during the whole 30 min of the OGD protocol. This integral was calculated by Igor routines (WaveMetrics). In some experiments we lose the recording before the end of OGD. In that case we measured only the time to the first peak amplitude and not I_OGD_ area. This explains why in the result section the number of cells in the statistics (n) is not always homogeneous. All drugs were added to the extracellular solution. Stocks of A-740003 (Art Molecule, Poitiers, France), CPA, 2-APB and TBOA were prepared in DMSO. Stocks of PPADS, TTX, APV, NBQX, MPEP, JNJ16259685 were dissolved in water. DIDS was dissolved in a solution of potassium bicarbonate.

### Calcium Imaging

Calcium imaging experiments were performed using the same patch clamp intracellular solution in which EGTA was substituted by the calcium sensitive dye Fluo-4 (100 μM, Molecular Probes-Invitrogen, France). After at least 20 min from breaking-in, the morphology of the cell was visualized and the presence of radial processes confirmed the electrophysiological identity of Bergmann cells. Labeled processes were focused in the optical field at a certain distance from the soma and they were illuminated at a single excitation wavelength (475 ± 40 nm). Excitation light coming from a 100W Xenon lamp, was gated by an electromechanical shutter (T132 Uniblitz). Calcium sensitive fluorescence changes were collected using a ×63 water-immersion objective, filtered by a barrier filter at 530 ± 50 nm (dichroic mirror 500 nm), recorded using a CCD camera (Coolsnap see, Photometrics) and triggered by the Software Metavue. Individual images were recorded every 10 s with an exposure time of 75 ms. A stable fluorescence baseline was required to perform the experiment and it was tested for at least 10 min before the OGD protocol. For the analysis, two regions were selected outside the loaded cell in order to define the background fluorescence and 4–6 regions of interest (ROIs) were chosen on Bergmann glia processes. The mean background was then subtracted from the ROIs and the relative fluorescence variation (ΔF/F) was calculated and expressed in percentage. In this way, at image “i”, ΔF_i_/F_0i_ = [(F_i_ − F_i0_)/F_i0_]*100, where F_i_ is the fluorescence at image “i” and F_i0_ the basal fluorescence measured before OGD. ΔF_i_/F_0i_ obtained for each ROI are then averaged in order to obtain for each recorded cell the temporal evolution of the mean fluorescence variation. On this type of function, the peak of the ΔF/F and the time to peak was measured and averaged among different cells. Moreover, in experiments with Ca^2+^-free extracellular solution or 2-APB, in order to quantify the ΔF/F in a late phase of OGD (22–30 min), we calculated the average fluorescence in that “plateau” phase and compared it to OGD in control conditions.

It is important to notice that after 7–10 min of OGD, the cerebellar tissue swelled (Hamann et al., 2005) rendering the analysis of calcium imaging experiments particularly difficult.

### Ion-Sensitive Microelectrode Recordings

The K^+^-sensitive microelectrodes were made according to the procedure used by Chever (Chever et al., 2010). Briefly, double-barreled electrodes were sylanized with dimethylchlorosilane, dried at 120°C for 2 h, and the tip of sylanized compartment was filled with the Potassium ionophore I-cocktail B (Sigma-Aldrich) and then with a solution of KCl at 0.2 M. The other barrel was filled with normal BBS solution for the recording of extracellular field potentials. Using an ion-sensitive amplifier (ION-01M, NPI, Germany), we recorded both the potential at the reference barrel and at the K^+^-microelectrode and it was also possible to record the substraction of these two signals in order to obtain the potential correlating exclusively with the [K^+^]_e_. The microelectrode was calibrated in BBS solution at different KCl concentrations (4.15 mM, 8 mM, 20 mM, 60 mM, 200 mM). Only K^+^-microelectrodes that provided stable recordings at every calibration solution change and that display voltage shifts of 58 mV for an increase in K^+^ concentration of 10 mM were used (Voipio et al., 1996). In order to convert the voltage signal to [K^+^]_e_, we used the Nernst equation.

### Statistics

Data were collected with the software Elphy (G. Sadoc, France). For analysis, sampling frequency was 2 kHz for recordings of spontaneous activity. Data analysis was performed off-line by using Clampfit (Axon Instruments) and Igor (WaveMetrics). Results are presented as mean ± SEM and statistical significance was set at 0.05 using the Student’s *t*-test or non-parametric (Mann-Whitney or Wilcoxon rank test) tests when samples were too small (*n* < 10) to verify the normal distribution; *n* indicates the number of cells included in the statistics.

## Results

### Bergmann Glia Electrophysiological Response to Ischemia

**Figure 1:**
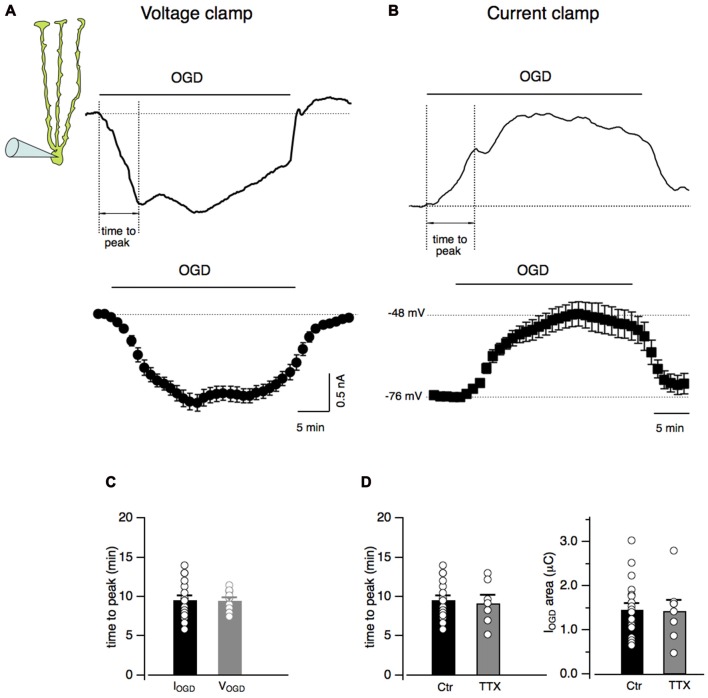
Oxygen and glucose deprivation (OGD) induces inward currents and membrane depolarization in Bergmann glial cells. **(A)** Current response of a Bergmann cell to 30 min of OGD (I_OGD_). The membrane potential is held at −70 mV. At the bottom, current traces from several Bergmann cells (*n* = 19) are averaged every minute. **(B)** Representative current clamp recording (V_OGD_) from a Bergmann cell during OGD. No current was injected in these experiments. Note that Bergmann glia depolarizes gradually during OGD while, in the post-OGD phase, the recovery toward the baseline membrane potential is faster. The time-dependent changes in membrane potential from *n* = 12 Bergmann cells during OGD is shown at the bottom. **(C)** Time intervals between the beginning of the OGD and the first peak of the response (dashed lines, “time to peak” in **(A,B)** are indicated for both I_OGD_ (*n* = 23) and V_OGD_ (*n* = 12; *P* = 0.88)). **(D)** The time to peak (Ctr, *n* = 23 and TTX, *n* = 7; *P* = 0.86, right) and the electrical charge underlying I_OGD_ (Ctr, *n* = 19 and TTX, *n* = 8; *P* = 0.93, left) are reported in control and in the presence of TTX (1 μM) for each recorded cells: there is no statistical difference between the two cell populations.

Bergmann cells were identified by the localization of their small-sized cell bodies in the Purkinje cell layer and by their unmistakable electrophysiological properties consisting in a low input resistance (12.7 ± 0.3 MΩ, *n* = 21) and a hyperpolarized membrane potential (−75.6 ± 1.0 mV, *n* = 21; not shown; Clark and Barbour, 1997). In order to study Bergmann glia response to *in vitro* ischemia, acute cerebellar slices underwent OGD from the extracellular solution. During 30 min of OGD protocol, Bergmann glia developed a progressive inward current that rapidly recovered to baseline in the post-OGD phase (Figure 1A). In current clamp experiments, the effect of OGD consisted into membrane depolarizations with a maximal value of 26.9 ± 4.1 mV (*n* = 12) and into a rapid repolarizing phase during the return to control solution (Figure 1B). In voltage clamp as well as in current clamp mode, the Bergmann glia response to OGD presented a first peak that was used here to measure the “time to peak”. As shown in Figure 1C, the current (I_OGD_) and the voltage (V_OGD_) responses to OGD have similar rise time kinetics (9.4 ± 0.5 min, *n* = 23 for I_OGD_ vs. 9.5 ± 0.4 min, *n* = 12 for V_OGD,_ *P* = 0.88). We decided to continue the present study in voltage clamped Bergmann cells and to characterize the OGD-induced current by calculating the total electrical charge underlying I_OGD_ (see “Materials and Methods” section, mean value: 1.5 ± 0.1 μC, *n* = 19, Figure 1B) and by the time to the first peak (9.4 ± 0.5 min, *n* = 23, Figures 1C,D).

Among the complex consequences of OGD, membrane potential depolarizations may induce a massive release of neurotransmitters. In order to check whether action potential firing may be responsible for I_OGD_, experiments were performed in the presence of TTX (1 μM) to prevent Na^+^-dependent action potential generation (Figures 1A,B). No significant changes were observed in I_OGD_ charge (0.14 ± 0.02 μC, *n* = 8, *P* = 0.93) or time to peak (9.2 ± 1.0 min, *n* = 7, *P* = 0.86) indicating that neuronal firing in the cerebellar slice does not contribute to Bergmann response to OGD. For this reason, the experiments were pursued without TTX.

### OGD Induces Intracellular Calcium Increases in Bergmann Glia

Astrocytes are considered non-excitable cells whose physiological functions and communication with other cells rely on increases in intracellular calcium. Bergmann cells are not an exception of the rule and exhibit spontaneous Ca^2+^ fluctuations both *in vitro* and *in vivo* (Hoogland and Kuhn, 2010). Therefore Ca^2+^ changes were studied during OGD in Bergmann glia processes.

**Figure 2:**
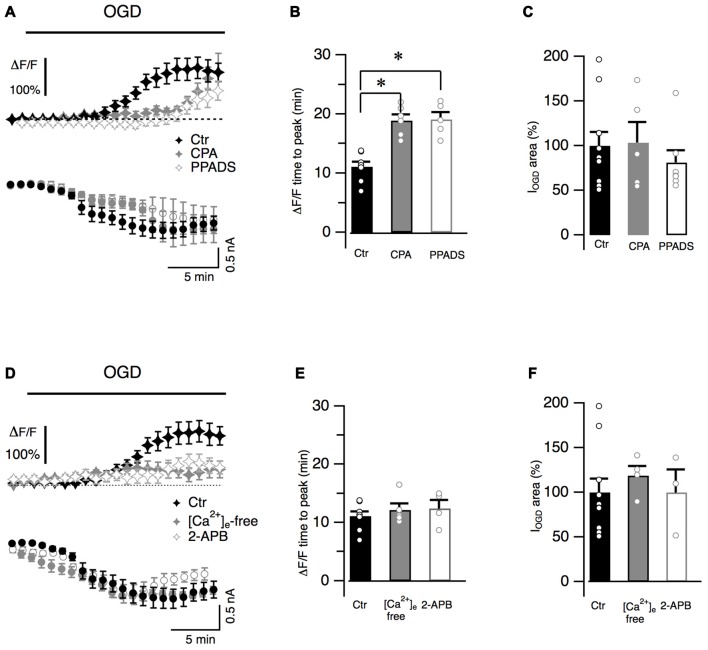
Bergmann glia Ca^2+^ raises during OGD are mediated by Ca^2+^ release from internal stores and Ca^2+^ entry from extracellular space. **(A)** Bergmann glial cells are loaded with Fluo4 (100 μM) and changes in fluorescence are measured in radial processes during OGD. Averaged ΔF/F values are plotted as a function of time in Ctr (*n* = 11), after treatment with CPA (20 μM), a blocker of intracellular Ca^2+^ stores refilling (*n* = 7) or with PPADS (100 μM), a broad-spectrum inhibitor of P2 receptors (*n* = 8). CPA and PPADS delayed the onset of intracellular Ca^2+^ increase (top) without affecting the onset of I_OGD_ (bottom). **(B)** Quantification of the effects of CPA (*P* = 0.002, *n* = 6) and PPADS (*P* = 0.0034, *n* = 5) on the kinetics of Ca^2+^ raises. **(C)** Mean and individual values of I_OGD_ area in control (*n* = 11), CPA (*n* = 5, *P* = 0.59) and in the presence of PPADS (*n* = 7, *P* = 0.12). **(D)** Extracellular Ca^2+^-free solution (+EGTA 5 mM, *n* = 9) or 2-APB (100 μM, *n* = 7), a blocker of store operated Ca^2+^ entry, dramatically reduces OGD-induced Ca^2+^ transients observed during OGD (Ctr, *n* = 11). **(E)** The time to the fluorescence peak is not affected by these treatments (*P* = 0.88, *n* = 5 for Ca^2+^-free solution and *P* = 0.27, *n* = 4, for 2-APB when compared to control (*n* = 8)). Note that the inward current dynamics **(D)** and the electrical charge **(F)** are not affected by the absence of extracellular Ca^2+^ (*P* = 0.51, *n* = 4) nor by 2-APB (*P* = 0.73, *n* = 3). *P < 0.005.

Cytosolic calcium increased during OGD and gradually reached a maximal value (ΔF/F = 140.1 ± 11.1%, *n* = 11, Figure 2A) that persisted throughout the entire duration of OGD protocol. To better characterize Ca^2+^ dynamics, the time from the OGD onset and the peak of fluorescence was measured for each recorded cell (time to peak: 11.0 ± 0.8 min, *n* = 8, Figure 2B). In order to investigate whether Ca^2+^ originates from intracellular Ca^2+^ stores, slices were incubated with CPA (20 μM), a blocker of SERCA pumps responsible for calcium store refilling. CPA crucially increased the latency of the calcium response (*n* = 7, *P* = 0.009; Figures 2A,B) while the maximal ΔF/F value was not statistically different from control values (to 168.7 ± 51.9% of the control, *n* = 6, *P* > 0.05). Activation of P2Y purinergic receptors can mobilize Ca^2+^ from internal stores in Bergmann glia processes (Beierlein and Regehr, 2006; Piet and Jahr, 2007). To determine whether these receptors were involved in Ca^2+^ increases during OGD, the effect of PPADS (100 μM), a broad-spectrum antagonist of purinergic receptors, was studied. Similarly to CPA, PPADS significantly increased the latency of the fluorescence peak (*P* = 0.0034, Figures 2A,B) and no Ca^2+^ increases were observed during the first 14 min of OGD protocol (ΔF/F = −0.2 ± 3.1% of the control, *n* = 7, *P* = 0.0016). The peak of the ΔF/F however was only marginally affected by the antagonist (to 79.18 ± 18.8% of the control, *n* = 5, *P* > 0.05). These data suggest that in the early OGD phase, P2Y receptors are activated and trigger Ca^2+^ release from internal stores. Interestingly, this calcium increase does not seem to be correlated to membrane current because neither CPA nor PPADS changed I_OGD_ onset (Figure 2A) or area (Figure 2C).

The effect of Ca^2+^-free extracellular solution was next explored on OGD Ca^2+^ fluctuations. Application of a nominally Ca^2+^ free solution (supplemented with EGTA 5 mM) reduced the basal fluorescence in Bergmann glia (by 38.5 ± 5.8%, *n* = 9, not shown). When OGD protocol was performed, the overall Ca^2+^ response was reduced when compared to the control (Figure 2D). The fluorescence raised with a latency comparable to control condition (Figure 2E) but the maximal fluorescence variation was only 47.9 ± 23.6% of the control (*n* = 5, *P* = 0.05, Figure 2D) suggesting that the presence of Ca^2+^ ions in the extracellular medium is fundamental for internal store refilling. Moreover after reaching a peak, the intracellular Ca^2+^ concentration significantly decreased (to 12.4 ± 13.3% of the control, *n* = 9, *P* = 0.004, not shown) indicating that in late OGD period (from 22 to 30 min), Ca^2+^ enters Bergmann processes from the extracellular space.

Store-operated Ca^2+^ channels are normally activated in Bergmann glia following depletion of Ca^2+^ stores (Singaravelu et al., 2006). We tested the possibility that these Ca^2+^ channels were activated during OGD by using 2-APB (100 μM) that efficiently inhibits these conductances in Bergmann glia (Singaravelu et al., 2006). Similarly to results obtained in Ca^2+^-free condition the mean maximal fluorescence was reduced to 59.6 ± 16.1% of the control with 2-APB (*n* = 5, *P* = 0.05, Figure 2D) and in the late OGD period (from 22 to 30 min) the mean ΔF/F was reduced to 25.1 ± 4.4%, of the control (*n* = 7, *P* = 0.02,) indicating a possible involvement of store-operated Ca^2+^ entry during *in vitro* ischemia. Again, Ca^2+^ ion charge was not implicated in I_OGD_ because I_OGD_ dynamics (Figure 2D) and amplitude (Figures 2D,F) were not affected by depletion of extracellular Ca^2+^.

These results all-together show that OGD induces a long-lasting intracellular Ca^2+^ increase in Bergmann glia that is mediated by both Ca^2+^ mobilization from stores and Ca^2+^ entry from the extracellular space. Moreover Ca^2+^ ion charges are not involved in the generation of I_OGD_ opening the question of the identification of the neurotransmitters involved in this electric current.

### Glutamate Receptors and Transporters Are Not Playing a Major Role in Bergmann Glia Responses to OGD

It has been shown that during ischemia, extracellular glutamate concentration increases dramatically in the cerebellum through both Ca^2+^-dependent vesicular release (Hamann et al., 2005) and Ca^2+^-independent mechanisms (Hamann et al., 2005; Beppu et al., 2014). As a consequence of this intense glutamate release, Purkinje neurons endure a severe anoxic depolarization through the activation of AMPA receptors (Hamann et al., 2005).

**Figure 3:**
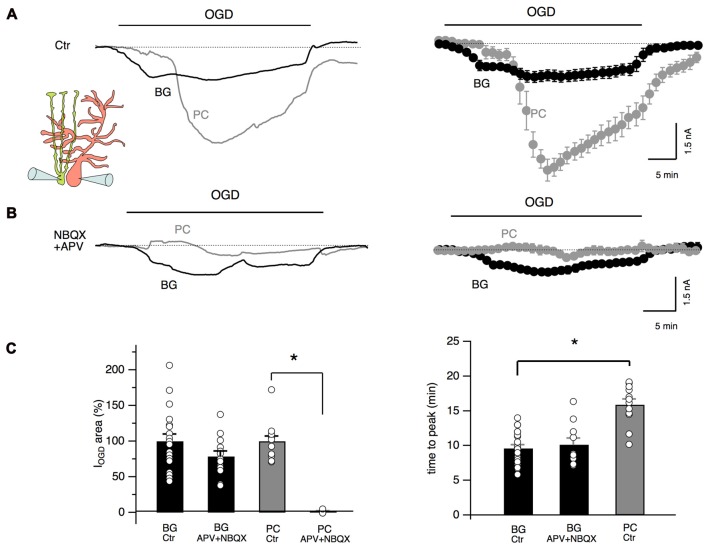
Glutamate differently affects Purkinje neurons and Bergmann glia. **(A)** Double, simultaneous patch clamp recordings of a Bergmann glial cell and a Purkinje neuron during OGD. Mean traces are shown at the right (*n* = 6). Note the difference in current dynamics, amplitude and post-OGD phase in the two cell types. **(B)** NBQX (25 μM) and APV (50 μM) drastically inhibit OGD-induced currents in Purkinje neurons while little effect is observed in I_OGD_ of Bergmann cells (*n* = 5). **(C)** Left: quantification of the electrical charge calculated in control or in the presence of ionotropic glutamate receptor blockers in Bergmann glial cells (*n* = 19 and *n* = 13 respectively, *P* = 0.13) and Purkinje neurons (*n* = 10 and *n* = 4 respectively, *P* = 0.0001). Right: the I_OGD_ time to peak is significantly delayed in Purkinje neurons (*n* = 19) when compared to Bergmann glia (*n* = 12, *P* = 0.0001). APV + NBQX do not change significantly the time to peak of the Bergmann glia I_OGD_ (*n* = 10, *P* = 0.47). *P < 0.005.

To test the possibility that glutamate release during cerebellar ischemia is also responsible for Bergmann cell responses, we performed double patch clamp recordings of Bergmann glia and Purkinje neurons during OGD protocol with or without antagonists of AMPA/kainate and NMDA receptors. As shown in Figure 3A, the temporal evolution of Bergmann cell and Purkinje cell currents during OGD is substantially different: at the beginning, Purkinje neuron holding current remained stable (or, in some cells, assumed outward values: 225 ± 54 pA, *n* = 10) while in Bergmann cell, I_OGD_ gradually developed as an inward current. Then, Purkinje cells presented a rapid and huge inward current (mean peak current: −5.7 ± 0.5 nA, *n* = 6) that reflect the “anoxic depolarization” previously reported by other teams (Hamann et al., 2005; Brady et al., 2010; Mohr et al., 2010). There was a delay of 16.9 ± 0.8 min (*n* = 6) from the start of OGD protocol and Purkinje cell peak current while for Bergmann glia the first I_OGD_ peak appeared significantly earlier (9.0 ± 0.9 min, *n* = 6, *P* = 0.0006, Figure 3C). In the post-OGD phase, the Purkinje cell current recovered only partly while Bergmann cell current completely returned to the baseline (Figure 3A). When we performed paired recordings in the presence of NBQX (25 μM) and APV (50 μM), the OGD-induced inward current was almost totally abolished in Purkinje neurons but we were surprised to observe that Bergmann cell I_OGD_ was only slightly affected by these antagonists (Figure 3B). These results were confirmed by single-cell patch clamp experiments in the presence of these blockers that indicated a reduction to 78.6 ± 7.7% of the control for Bergmann glia I_OGD_ area (*n* = 13, *P* = 0.12; Figure 3C) and to 1.3 ± 1.3% of the control for Purkinje cell OGD-induced current (*n* = 5, *P* = 0.01; Figure 3C). Furthermore, Bergmann glia Ca^2+^ dynamics were not significantly affected by ionotropic glutamate receptor antagonists (early phase: 64.1 ± 15.5% of the control, *P* = 0.08; late phase: 117.4 ± 13.4% of the control *P* = 0.2, *n* = 4, not shown) confirming that these receptors are poorly activated in Bergmann glial processes during OGD.

**Figure 4:**
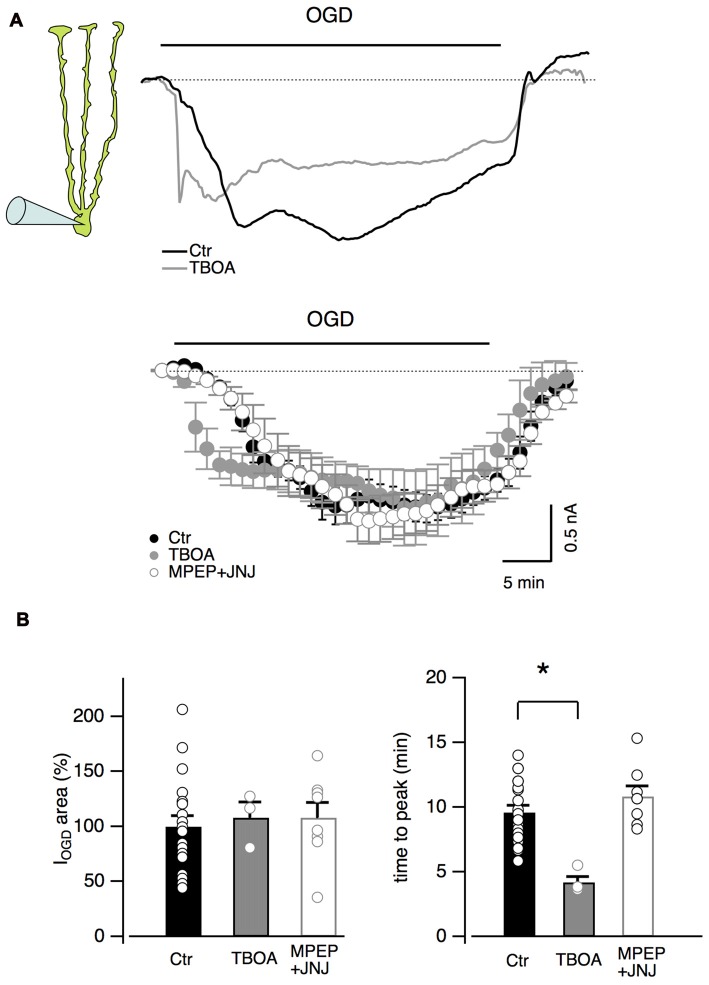
Inhibition of glutamate transporters accelerates OGD kinetics in Bergmann glia. **(A)** Top: examples of Bergmann glia currents in control and in the presence of TBOA (100 μM), an inhibitor of glutamate transporters. Bottom: mean traces in control (*n* = 19), in presence of TBOA (*n* = 4) or with group I metabotropic glutamate receptor blockers (MPEP 5 μM + JNJ16259685 1 μM, *n* = 8). **(B)** Neither TBOA (*P* = 0.88, *n* = 4) nor MPEP + JNJ16259685 (*P* = 0.66, *n* = 8) significantly affect the OGD-induced current charge (left) while, TBOA significantly decreases the time to peak of OGD-induced currents (*n* = 4, *P* = 0.001, right). *P < 0.005.

Other inhibitors of the glutamatergic system were also tested on Bergmann glial cells (Figure 4). The antagonists of type I metabotropic glutamatergic receptors, MPEP (5 μM) and JNJ16259685 (1 μM) did not significantly affect the OGD-induced current (*P* = 0.66, *n* = 8, Figures 4A,B) or time to the first peak (*P* = 0.15, *n* = 8, Figure 4B) while the blocker of glutamate transporters, TBOA (100 μM), significantly reduced the onset of I_OGD_ (*P* = 0.001, Figures 4A,B) leaving the mean amplitude unchanged (Figure 4B, *P* = 0.88). A similar effect of TBOA has been observed in Purkinje neurons during OGD (Beppu et al., 2014).

All together, these experiments indicate that glutamate released during OGD totally account for the depolarizing current observed in Purkinje neurons but it has only minor effects on I_OGD_ and Ca^2+^ increases observed in Bergmann glia. This pharmacological result together with distinct I_OGD_ kinetics for Bergmann glia and Purkinje neurons, suggest that glia cells are not always merely following neuronal reactions.

### Bergmann Glia Ionotropic P2X7 Receptors Are Not Activated during OGD

**Figure 5:**
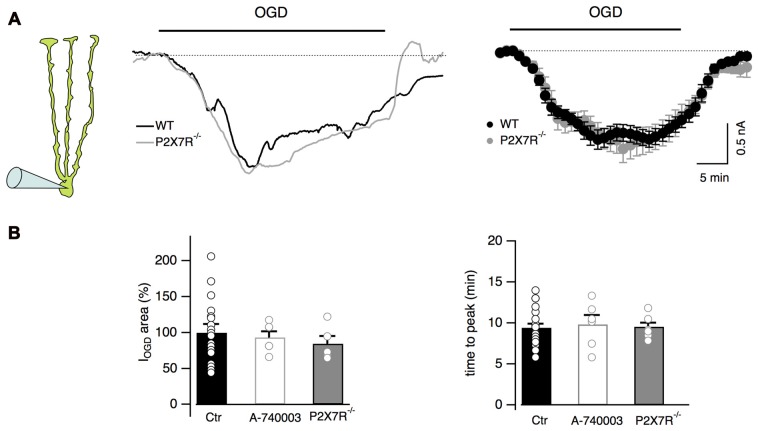
P2X7 receptor activation is not observed during OGD. **(A)** Representative currents from a Bergmann glial cell in wild type and P2X7R^−/−^ mice. Mean currents are shown at the right (*n* = 19 and *n* = 8 from wild type and P2X7R^−/−^ mice respectively). **(B)** No statistical differences are observed in the electrical charge or in the time to peak of I_OGD_ among WT, P2X7R^−/−^ mice and cells from wild-type mice treated with the P2X7R antagonist, A-740003 (10 μM). For I_OGD_ charge: *n* = 19 in WT, *n* = 6 in A-740003 (*P* = 0.4) and *n* = 5 in P2X7R^−/−^ (*P* = 0.91); for time to peak: *n* = 23 in WT, *n* = 6 in A-740003 (*P* = 0.68) and *n* = 7 in P2X7R^−/−^ (*P* = 0.31).

It has been reported that during ischemia extracellular ATP concentration increases (Braun et al., 1998; Melani et al., 2005) leading to activation of both P2Y and P2X7 receptors in some brain areas (Domercq et al., 2010; Arbeloa et al., 2012; but see also Leichsenring et al., 2013). Our Ca^2+^ imaging results indicate that Bergmann cell P2Y receptors are activated during OGD (Figure 2) suggesting that ATP can be released in the cerebellar cortex during ischemic conditions. We therefore explored the possibility that P2X7 receptors were also activated during OGD and could be involved in Bergmann depolarization. For this purpose, the effects of OGD were tested in Bergmann glia from P2X7R^−/−^ mice. No differences were observed between WT and P2X7R^−/−^ mice (I_OGD_ = 1.4 ± 0.2 μC, *n* = 5 in P2X7R^−/−^ mice, *P* = 0.91 when compared to control, Figures 5A,B), a result that was confirmed by using the selective P2X7 receptor antagonist A-740003 (10 μM) in wild type mice (I_OGD_ = 1.6 ± 0.1 μC, *P* = 0.4, *n* = 6; Figure 5B).

### Extracellular K^+^ Concentration Increases during Cerebellar OGD

**Figure 6:**
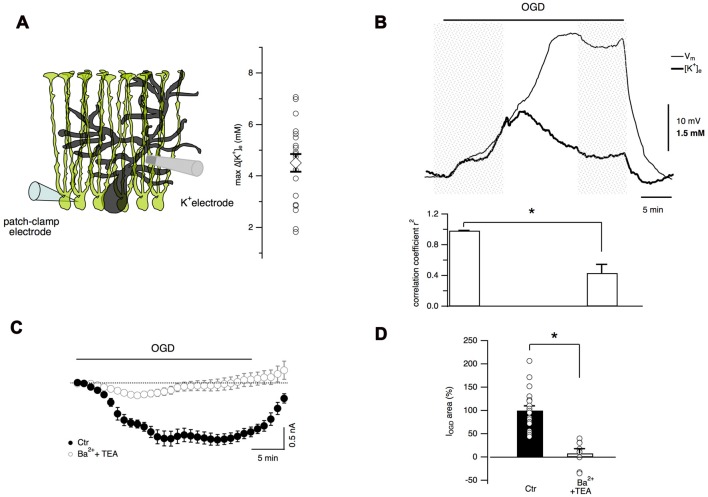
Extracellular K^+^ accumulation during OGD partially account for Bergmann cell depolarization. **(A)** Extracellular K^+^ concentration is measured through an ion-sensitive microelectrode placed in the molecular layer. Maximal values of [K^+^]_e_ variations recorded during OGD are reported in the plot (*n* = 22). **(B)** An example of simultaneous recordings of [K^+^]_e_ changes and Bergmann glia membrane potential during OGD (top). Bottom: during the first 10 min of OGD protocol, the membrane potential and [K^+^]_e_ increase concomitantly revealing high degree of correlation (*n* = 7) while after this time, [K^+^]_e_ decreases and membrane depolarization increases further. The *P* value for the histogram data analysis is *P = 0.02, Wilcoxon Signed-rank test. **(C)** Mean currents recorded in control (*n* = 19) and in the presence of 5 mM Ba^2+^ and 10 mM TEA (*n* = 8). **(D)** These K^+^ channel inhibitors significantly reduce the electrical charge of Bergmann glia I_OGD_ (*P = 0.0002).

It has been well documented that, due to the abundance of K^+^ channels, astrocyte membrane potential closely follows the [K^+^]_e_ variations (Walz, 2000). During cerebral ischemia, [K^+^]_e_ increases dramatically and astrocytes may play a key role in K^+^ homeostasis through their K^+^ transporters, ion channels and extensive gap junction coupling (Leis et al., 2005). Therefore it was fundamental to measure extracellular K^+^ changes during cerebellar OGD through ion-sensitive electrodes placed in the molecular layer (Figures 6A,B). With this technique, a gradual increase in [K^+^]_e_ was observed during OGD (maximal [K^+^]_e_ increase 4.5 ± 0.3 mM, *n* = 20 slices, Figure 6A). In an attempt to correlate K^+^ concentration changes and membrane potential in Bergmann glia, ion-sensitive electrode measurements were performed simultaneously with Bergmann glia current-clamp recordings (Figure 6B). During the first 10 min of OGD, Bergmann glia membrane depolarization and [K^+^]_e_ increase were tightly coupled showing a high degree of correlation (correlation coefficient *r*^2^ = 0.984 ± 0.003, *n* = 7). However, after reaching a peak value, [K^+^]_e_ decreased slowly until a plateau value of 1.04 ± 0.34 mM above the baseline (at 30 min OGD, *n* = 6) while the membrane potential of the glial cell depolarized to a steady state value of −47.9 ± 4.8 mV (from a mean resting potential of −76.73 ± 1.16 mV, *n* = 7) revealing that in the late OGD period, Bergmann membrane potential and [K^+^]_e_ variations are less correlated (*r*^2^ = 0.37 ± 0.11, *n* = 7, *P* = 0.02, Wilcoxon signed-rank test, Figure 6B) implying that another mechanism comes into play.

To confirm the activation of K^+^ conductances during OGD, experiments were carried out in the presence of barium (5 mM) and TEA (10 mM). As shown in Figures 6C,D, these inhibitors almost completely abolished I_OGD_ (93.2 ± 8.8%, *P* = 0.0002, *n* = 8). The effect of barium and TEA on [K^+^]_e_ dynamics has not been studied because these drugs had an inhibitory action on the K^+^ ionophore used for ion-sensitive recordings, making this type of experiment unachievable (unpublished observations).

However, all together these data indicate that the increase in [K^+^]_e_ during OGD induces a progressive membrane depolarization in Bergmann cells and that I_OGD_ is likely due to K^+^ accumulation in the extracellular space suggesting a possible implication of Bergmann glia in K^+^ ion buffering during cerebellar ischemia.

### DIDS-Sensitive Anion Channels Are Activated during OGD

**Figure 7:**
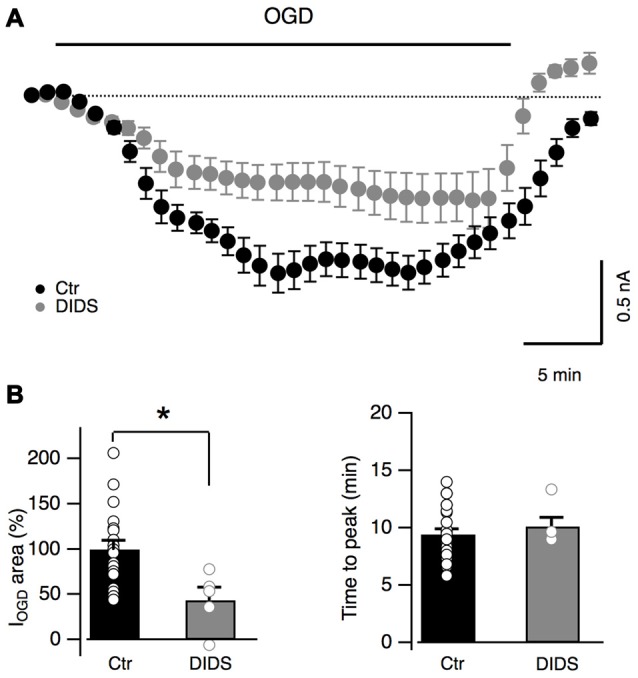
DIDS partially blocks OGD-induced currents in Bergmann glia. **(A)** Average currents recorded during OGD with (*n* = 7) or without (*n* = 19) a non-specific blocker of anion channels DIDS (1 mM). **(B)** Average changes of I_OGD_ area and time to peak in control (*n* = 19) and in the presence of DIDS (*n* = 5). *P* = 0.017 for I_OGD_ area and *P* = 0.33 for I_OGD_ time to peak. *P < 0.05.

It has been recently shown that Bergmann glia undergo cytosolic acidification during OGD inducing the activation of anionic channels sensitive to DIDS (Sasaki et al., 2012; Beppu et al., 2014). These anionic channels have been proposed to be permeable to glutamate during cerebellar OGD but the effect of their activation on Bergmann glia has not been reported (Beppu et al., 2014). In order to investigate whether these channels are involved in Bergmann response to OGD, DIDS (1 mM) was added to the extracellular solution. DIDS induced an inward current in all Bergmann cells tested (mean current value: 387 ± 60 pA, *n* = 7, not shown) and significantly reduced I_OGD_ (to 43.7 ± 14.2% of the control, *n* = 5, *P* = 0.017, Figures 7A,B) without changing the time to the first peak (10.1 ± 0.8 min, *n* = 5, *P* = 0.33 compared to the control condition, Figure 7B). These results indicate that anion outflow through DIDS-sensitive channels is implicated in Bergmann glia I_OGD_.

## Discussion

**Figure 8:**
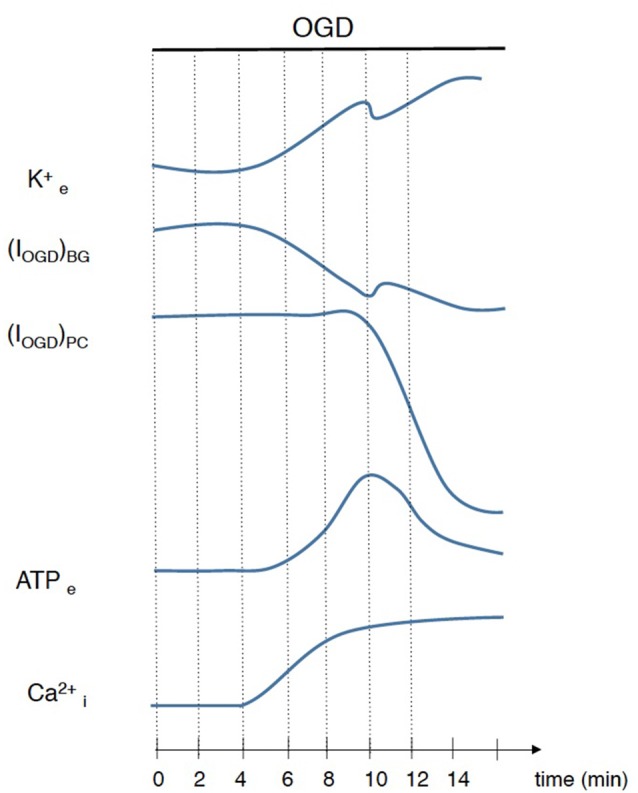
Schematic illustration of events that occur during ischemia simulated by OGD. Interruption of ATP production leads to an imbalance of ionic gradients resulting in an accumulation of K^+^ in extracellular space and consequent Bergmann glia depolarization. This disruption of ion homeostasis depolarizes cerebellar neurons exacerbating glutamate release that, with several minutes of delay, induces a huge depolarization in Purkinje cells. ATP extracellular concentration is also increased during OGD and is responsible, at least in part, for Ca^2+^ rises in Bergmann glial cells.

In this study we characterized both the electrical and the calcium responses of cerebellar Bergmann glia in ischemia-like conditions induced by an OGD protocol. The OGD protocol has indeed been shown to reproduce closely the principal effects of *in vivo* ischemic attacks (Rossi et al., 2000), and thus likely represents an appropriate model for reproducing cerebellar ischemia *in vitro*. Our results show that during OGD episodes, Bergmann glial cells depolarize and display prolonged intracellular Ca^2+^ increases. These complex responses are generated by the temporal succession of multiple events including an increase in extracellular K^+^ concentration, neurotransmitter release, Ca^2+^ mobilization from internal stores and Ca^2+^ entry from the extracellular space (Figure 8).

### Bergmann Glia Behave as K^+^ Sensors during Early OGD

K^+^ accumulations in the extracellular space are hallmark events accompanying cerebellar ischemia (Kraig et al., 1983). The loss of metabolic ATP associated with an ischemic event leads to progressive failure of ionic pumps, to the gradual loss of ionic gradients, and finally to K^+^ outflow from cells, and membrane depolarizations.

The dominant permeability of astrocytes to K^+^ endows them with exquisite sensitivity to [K^+^]_e_ changes, and with the specific capability of buffering activity-dependent variations of this ion’s concentration (MacAulay and Zeuthen, 2012). Bergmann cells are not an exception in this panorama. Consistently, extracellular K^+^ buffering by these glial cells has been found to modulate Purkinje cell firing patterns (Wang et al., 2012).

Our experiments demonstrate that the increases of [K^+^]_e_ detected during the early phase of OGD (≈ first 10 min) are temporally correlated with Bergmann depolarizations. Their quantification (averaged [K^+^]_e_ increases amount to 4.5 mM) also allowed us to predict precisely membrane potential changes using the Nernst equation (theoretical: 18.9 mV vs. recorded: 17.8 ± 0.5 mV, *n* = 12), further confirming that potassium homeostasis is a decisive factor in determining Bergmann glia electrical properties also during pathological conditions.

We also found that application of the unspecific K^+^ channel blockers barium and TEA totally inhibits these depolarizing responses, consistently with their antagonistic effects on Bergmann glia passive conductances (Müller et al., 1994; Sasaki et al., 2012). The high concentrations required to achieve a complete block, though, do not allow us to identify unambiguously the channel types underling K^+^ accumulation and/or buffering during OGD. A role for several glial and/or neuronal K^+^ conductances such as two-pore domain K^+^ channels (Zhou et al., 2009; Benesova et al., 2012), voltage-dependent K^+^ channels (Gibor et al., 2004; Hibino et al., 2010) or inward rectifying Kir4.1 channels (Olsen et al., 2006; Tong et al., 2014), which are highly expressed in Bergmann glia (Higashi et al., 2001; Djukic et al., 2007), is thus conceivable.

In *in vivo* experiments, it has been reported that, following ischemia [K^+^]_e_ increases up to several tens of milliMoles in the cerebellum (Kraig et al., 1983) higher than what we report in this study. This discrepancy is probably due to the fact that acute slices represent a simplified model of what happens in the whole animal. The OGD protocol indeed mimics ischemia through a deprivation of O_2_ and glucose in the bathing medium, whereas in *in vivo* conditions ischemia is induced by a variety of whole animal manipulations like cardiac arrest caused by injections of high concentrations of potassium (Kraig et al., 1983). Furthermore, during our recordings, slices are continuously perfused (a procedure which cannot be avoided in order to maintain the physiological-like temperature of the preparation), and this certainly leads to extended washout of ions, neurotransmitters and other molecules released by cells into the extracellular space. Consistently, in some experiments we observed that [K^+^]_e_ increases are notably larger when slice perfusion is interrupted, thus further approaching *in vivo* conditions (data not shown).

### Possible Mediators of the Late Phase of Bergmann Glia I_OGD_

Potassium ions accumulation in the extracellular space can explain Bergmann cell depolarizations only during early OGD. Later during energy deprivation, our data indeed show that the membrane potential continues to depolarize while [K^+^]_e_ decreases, indicating that other mediators are implicated in the Bergmann cell electrical responses to ischemic events. None of the several distinct pharmacological blockers, which we examined, had a significant impact on the amplitude of I_OGD,_ with the exception of DIDS, a blocker of anionic conductances. This finding is compatible with recent data from other groups showing that these channels are involved in glutamate release from Bergmann glia during OGD (Beppu et al., 2014). Our data are also in line with the hypothesis that an important contribution to membrane depolarizations derives from the outflow of negative charges from cells, namely either glutamate or other anions, through volume-regulated channels activated by the cellular swelling accompanying OGD (Brady et al., 2010; our personal observations also indicate important cellular swelling during OGD). DIDS may inhibit both a large spectrum of anion channels such as ClC chloride channels (Blanz et al., 2007; Jeworutzki et al., 2012) and volume-regulated anion channels (Cavelier and Attwell, 2005; Liu et al., 2009), and also anion transporters such as the Na^+^/HCO_3_^−^ cotransporter (Tauskela et al., 2003) and the Cl^−^/HCO_3_^−^ exchanger (Kobayashi et al., 1994; Hentschke et al., 2006).

### ATP, but Not Glutamate, Mediates Bergmann Glia Responses to OGD

Importantly, our study highlights major differences between the mechanisms mediating neuronal and glial responses to OGD in the cerebellar cortex (Figure 8).

In particular, glutamate release is the principal responsible for anoxic depolarizations of Purkinje neurons, whereas our data clearly indicate that it does not contribute to the currents and the Ca^2+^ transients developing in Bergmann glia during ischemia. This is a highly unexpected finding because Bergmann glia express Ca^2+^-permeable AMPA receptors (Geiger et al., 1995) that are normally activated by glutamate originating from both parallel and climbing fibers in control conditions (Clark and Barbour, 1997; Matsui and Jahr, 2003). A possible explanation for this apparent contradiction may derive from the specific localizations of glia AMPA receptors at ectopic release sites (Matsui et al., 2005) where glutamate release follows different rules with respect to active zones (Matsui and Jahr, 2003; Bellamy and Ogden, 2005). Ectopic sites indeed seem to lack the fast vesicle recycling mechanisms that normally operate at synaptic sites (Balakrishnan et al., 2011), leading to strong synaptic depression of parallel fiber transmission during either high frequency or prolonged low frequency stimulation (Bellamy and Ogden, 2005). It is therefore possible that the global increase in neuronal excitability observed during OGD may similarly produce depression of ectopic glutamate release and reduce activation of glial AMPA receptors during OGD.

ATP is another neurotransmitter putatively involved in cerebral responses to ischemia. Our results suggest that ATP is indeed released during OGD, and that it activates purinergic P2Y receptors in Bergmann cells. In the cerebellum, ATP may either be synaptically released from molecular layer fibers (Beierlein and Regehr, 2006; Piet and Jahr, 2007) depolarized by the high [K^+^]_e_ increases taking place during OGD, or originate from necrotic cells damaged by the ischemic protocol (Mohr et al., 2010). Astrocytes are also putative sources of extracellular nucleotides via activation of conductances such as the volume-regulated anion channels following ischemia-triggered cell swelling (Kimelberg et al., 2006; Hamilton and Attwell, 2010).

Here, we also show that PPADS, a broad-spectrum antagonist of P2 receptors, has specific delaying effects on the time course of Bergmann glia Ca^2+^ responses to OGD without affecting the amplitude of the concomitant depolarizing currents. This effect is most likely due to the inhibition of P2Y metabotropic receptors by PPADS. P2Y receptors are indeed high affinity ATP/ADP sensors (Fields and Burnstock, 2006) that can mobilize Ca^2+^ from Bergmann glia internal stores (Beierlein and Regehr, 2006; Piet and Jahr, 2007; Wang et al., 2012). In contrast, we have no evidence in favor of the activation of ionotropic P2X7 receptors (Habbas et al., 2011), which have a very low affinity for ATP (North, 2002; Young et al., 2007; Habbas et al., 2011) and whose role in brain ischemia is still debated following contrasting data obtained in the hippocampus and in the neocortex (Arbeloa et al., 2012; Leichsenring et al., 2013). Consistently with our data, previous studies have reported that ATP concentration increases in the extracellular space during an ischemic episode *in vivo* (Braun et al., 1998; Kharlamov et al., 2002; Pedata et al., 2016) and that PPADS significantly improves ischemic lesions in the cortex (Lämmer et al., 2006).

### Other Possible Channels Implicated in OGD-Mediated Effects on Bergmann Glia

It is important to mention that ion channels other than those mentioned so far may be involved in the responses of Bergmann glia to OGD. These conductances include hemichannels that have been proposed to participate to the membrane depolarization of hippocampal neurons during OGD (Thompson et al., 2006; Thompson, 2015) and Ca^2+^-permeable transient receptor potential (TRP) channels (Aarts et al., 2003; Weilinger et al., 2013). Bergmann glial cells are extensively coupled through gap junctions (Müller et al., 1996; Tanaka et al., 2008), nonetheless it seems unlikely that these channels mediate I_OGD_ in Bergmann glia as carbenoxolone (100 μM), an inhibitor of electrical connections, has no major effects on I_OGD_ in our conditions (data not shown). Regarding TRP channels, some TRP subtypes have been found in astrocytes and neurons of the cerebellar granule layer (Shibasaki et al., 2013), and in Purkinje cells (Zhou et al., 2014). Although there is no direct evidence supporting TRP channel expression in Bergmann glia, we cannot totally exclude the possibility that they intervene in OGD responses, also because of our calcium imaging results suggesting that part of the cytosolic Ca^2+^ increase during OGD is mediated by Ca^2+^ entry from the extracellular space. We used 2-APB to inhibit store-operated calcium entry (SOCE) that occurs in Bergmann glia (Singaravelu et al., 2006), however 2-APB is not specific for SOCE and it may also act on IP_3_ receptors (Maruyama et al., 1997) or TRP channel subtypes that mediate Ca^2+^ entry and cell death during ischemia (Aarts et al., 2003; Weilinger et al., 2013).

### Possible Roles for Bergmann Glia during Ischemia

Simultaneous patch-clamp recordings revealed precious temporal information about the time course of the responses to OGD of Bergmann glia and Purkinje neurons, further revealing important differences between these two cells, as follows: (1) Bergmann glia membranes depolarize gradually a few minutes following OGD onset, as a consequence of the increase in [K^+^]_e_. No depolarizing currents are observed in Purkinje neurons in this early phase, although the increase in the frequency of spontaneous postsynaptic currents recorded in Purkinje neurons (from 2.8 ± 0.3 Hz to 6.1 ± 0.7 Hz, *n* = 7, not shown) clearly demonstrates that network excitability is already enhanced at this stage; (2) large inward currents develop in Purkinje neurons only late after OGD onset (≈15 min), reflecting the accumulation of extracellular glutamate, whereas, as mentioned previously, neighboring Bergmann glia are poorly affected by this neurotoxic transmitter; and (3) finally, in the post-OGD phase, judging by electrophysiological criteria, Bergmann glial cells recover completely while Purkinje neurons display only either a partial, or no recovery at all.

Can we conclude for these reasons that Bergmann glial cells are more resistant to ischemia than Purkinje neurons? In this same direction, is the overall role of Bergmann glial cells detrimental or protective for neuronal function? On one side, in the cerebellum glutamate release from Bergmann glia has been found to be closely associated with intracellular acidifications during OGD, indicating that these cells might be implicated in neurotoxicity (Beppu et al., 2014). On the other hand, although we could not unambiguously identify a role for Bergmann glia in generating the excitatory drive that kills Purkinje cells during OGD, here we nonetheless found elements suggesting that Bergmann glia could participate to the uptake of K^+^ from the extracellular space, a function possibly protecting and supporting neurons.

In the future, it will be interesting to tackle this issue in conditions that selectively target glial cells. A pharmacological approach using fluorocitrate, a gliotoxin that under some conditions, selectively inhibit astrocyte metabolism (Hassel et al., 1992; Vance et al., 2015) may represent a valuable strategy to perturb Bergmann glia membrane potential and its K^+^ buffer capabilities. An alternative genetic approach would consist in using Kir4.1 knockout mice. Kir4.1 channels are glia-specific K^+^ conductances that are fundamental to maintain a hyperpolarized membrane potential in glial cells and thus they support an efficient extracellular K^+^ buffering (Chever et al., 2010). Therefore these mice could represent a good model to study Bergmann glia-Purkinje neurons interaction during OGD. However, the conditional knockout of Kir4.1 gene in astrocytes induces premature death in mice (Djukic et al., 2007) thus limiting experiments on mature animals (as performed in this article).

Clearly, more elements are needed to provide a response to the important question regarding the exact role of astrocytes during ischemia. We believe that, once obtained, this information will contribute importantly to the development of effective treatment strategies for individuals touched by this highly destructive event.

## Author Contributions

RH contributed to conception and design of the study via data acquisition, analysis/interpretation and revision of the manuscript; OC participated to data acquisition and contributed to the set up of ion-sensitive microelectrode technique; HD participated in interpretation of the data and revision of the manuscript. MG contributed to conception and design of the study, data interpretation and analysis, preparation/revision of the manuscript. All authors have approved the final version of the manuscript and qualify for authorship.

## Conflict of Interest Statement

The authors declare that the research was conducted in the absence of any commercial or financial relationships that could be construed as a potential conflict of interest.
